# DTI fiber-tracking parameters adjacent to gliomas: the role of tract irregularity value in operative planning, resection, and outcome

**DOI:** 10.1007/s11060-024-04848-3

**Published:** 2024-10-15

**Authors:** Daniele Armocida, Andrea Bianconi, Giuseppa Zancana, Tingting Jiang, Alessandro Pesce, Fulvio Tartara, Diego Garbossa, Maurizio Salvati, Antonio Santoro, Carlo Serra, Alessandro Frati

**Affiliations:** 1 Department of Neuroscience “Rita Levi Montalcini”, Neurosurgery Unit, University of Turin, Via Cherasco 15, Turin (TO), 10126 Italy; 2 IRCCS “Neuromed”, via Atinense 18, 86077 Pozzilli, IS Italy; 3 Human Neurosciences Department Neurosurgery Division, “La Sapienza” University, Policlinico Umberto 6 I, viale del Policlinico 155, Rome (RM), 00161 Italy; 4 Neurosurgery Unit, Università degli studi di Roma (Tor Vergata), Policlinico Tor Vergata (PTV), Viale Oxford, 81, 00133 Rome (RM), Italy; 5 Unit of Neurosurgery, Istituto Clinico Città Studi, Milan, Italy; 6 Department of Neurosurgery, Clinical Neuroscience Center, University Hospital Zurich, University of Zurch, Frauenklinikstrasse 10, CH-8091 Zurich, Switzerland

**Keywords:** Glioma, Neurosurgery, Tractography, DTI

## Abstract

**Purpose:**

The goal of glioma surgery is maximal tumor resection associated with minimal post-operative morbidity. Diffusion tensor imaging-tractography/fiber tracking (DTI-FT) is a valuable white-matter (WM) visualization tool for diagnosis and surgical planning. Still, it assumes a descriptive role since the main DTI metrics and parameters showed several limitations in clinical use. New applications and quantitative measurements were recently applied to describe WM architecture that surround the tumor area. The brain adjacent tumor area (BAT) is defined as the region adjacent to the gross tumor volume, which contains signal abnormalities on T2-weighted or FLAIR sequences. The DTI-FT analysis of the BAT can be adopted as predictive values and a guide for safe tumor resection.

**Methods:**

This is an observational prospective study on an extensive series of glioma patients who performed magnetic resonance imaging (MRI) with pre-operative DTI-FT analyzed on the BAT by two different software. We examined DTI parameters of Fractional anisotropy (FA mean, min-max), Mean diffusivity (MD), and the shape-metric “tract irregularity” (TI) grade, comparing it with the surgical series’ clinical, radiological, and outcome data.

**Results:**

The population consisted of 118 patients, with a mean age of 60.6 years. 82 patients suffering from high-grade gliomas (69.5%), and 36 from low-grade gliomas (30.5%). A significant inverse relationship exists between the FA mean value and grading (*p* = 0.001). The relationship appears directly proportional regarding MD values (*p* = 0.003) and TI values (*p* = 0.005). FA mean and MD values are susceptible to significant variations with tumor and edema volume (*p* = 0.05). TI showed an independent relationship with grading regardless of tumor radiological features and dimensions, with a direct relationship with grading, ki67% (*p* = 0,05), PFS (*p* < 0.001), and EOR (*p* < 0.01).

**Conclusion:**

FA, MD, and TI are useful predictive measures of the clinical behavior of glioma, and TI could be helpful for tumor grading identification and surgical planning.

## Introduction

Gliomas are the most common primary neoplasms of the central nervous system (CNS) in adults [1, 2], and their prognosis is influenced by the molecular profile [3], extent of surgical resection (EOR) [4] age [5], and performance status [6]. Realizing the true benefit of neurosurgical resection requires a balance between surgical cytoreduction and preservation of neurological function in the concept of “neuro-oncological balance” [1].

Magnetic resonance imaging (MRI) with diffusion tensor imaging-fiber tracking (DTI-FT) is becoming a standard imaging method to achieve the best surgical planning [7]. DTI-FT is used to visualize specific fiber bundles in the surroundings of brain tumors [8]. Gliomas may alter surrounding white matter (WM) tracts depending on the grading, surgical site, tumor, and edema volume [7–9]. For these reasons, DTI-FT assumes a descriptive role by lacking validated *quantitative* data and values useful for diagnosis and preoperative planning [10].

Recent studies [11, 12] diverted their attention to DTI-FT analysis to the periphery of the glioma, claiming that the brain adjacent tumor (BAT) area contained a considerable amount of altered fiber tracts with less infiltration and disruption. The BAT is currently defined as the region adjacent to the gross tumor volume, which contains signal abnormalities on T2-weighted or FLAIR sequences [12].

**Fig. 1:**
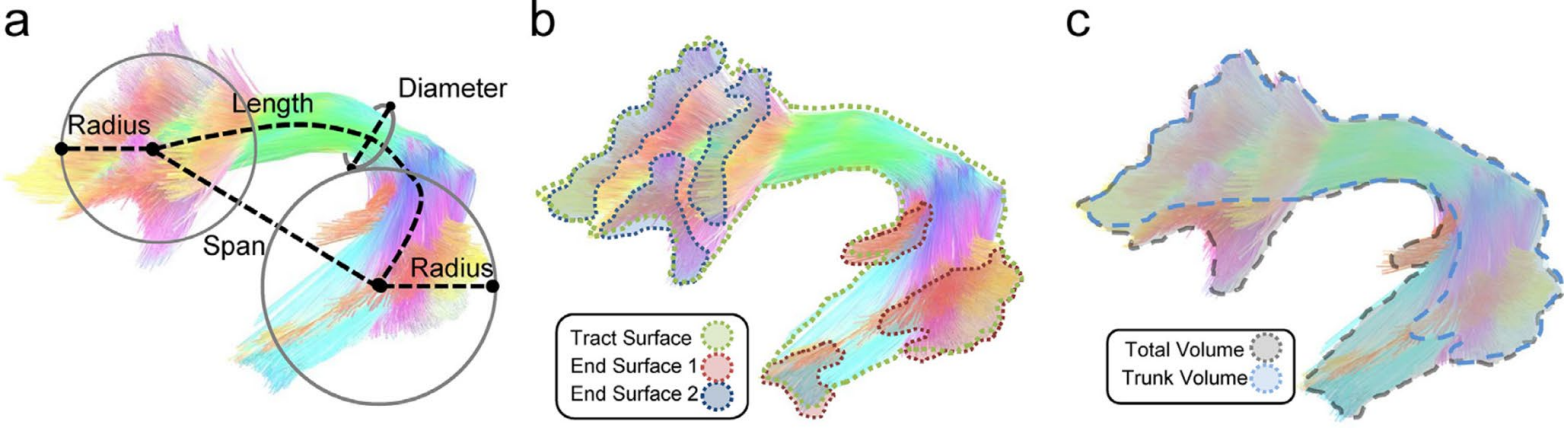
Shape analysis of a bundle described by Yeh FC [11]: (**a**) The length metrics include length, span, diameter, and radius of the innervation region. The length measures the length of the bundle trajectory, whereas the span measures the absolute distance between two ends of the bundle. The diameter estimates the average bundle diameter. The radius uses a circular model to estimate the coverage of the innervation regions. (**b**) The area metrics include total track surface area and area of the two end surfaces. (**c**) The volume metrics include total volume and trunk volume

In the BAT the main DTI metrics (fractional anisotropy, FA, and mean diffusivity, MD) are determined by a balance between factors that increase the degree of directionality of water diffusion (the “anisotropy”), such as high cellularity [13] and/or vascularization [14], and factors that decrease the degree of directionality, such as fiber destruction or infiltration [11]. Further, new quantitative measurements defined as “shape descriptors” were applied to investigate the shape characteristics of the human association pathways. Yeh FC [15] introduced a valuable quantification of the shape metrics and, specifically, the descriptor called “tract irregularity” (TI), defined as a numerical ratio between the surface area, diameter, and length of specific fibers (Fig. 1) that could offer a new option for WM analysis [15]. The diffusion metrics measured in the BAT promise to make DTI-FT a grading-predictive tool and a more precise aid in guiding glioma resection.

The objective of the present study is to test the validity of the main quantitative parameters of DTI-FT to establish the grading and behavior of a series of glioma patients. We compare glioma patients’ clinical, morphological, molecular, and outcome parameters to establish the predictive values of FA and MD with the combined use of the TI shape descriptor, measured in the BAT.

## Methods

This prospective observational study was performed on a surgical series of glioma patients treated in three neurosurgical units. Consensus about diagnosis, treatment, and related information was obtained under written informed consent approved by our Institution’s Principal Institutional Review Board (IRB: 6961, prot. 0296/2023).

This study adhérés to PROBE 2023 guidelines for reporting observational studies.

### Data availability

The original dataset is available from the corresponding author upon reasonable request.

### Population study

All subjects with radiological diagnosis suggestive of glioma and candidates for surgery were enrolled from March 2018 to December 2022, with the following inclusion criteria:

- Adult patients with unilateral surgical suspected glioma with no history of inflammatory or degenerative brain disease candidate for surgery;
- Patients with histological diagnosis of glioma, following the World Health Organization (WHO) 2021 [16] classification of brain tumors with a minimum of 12 months of follow-up;
- Performance status measured using Karnofsky performance scale (KPS) > 70;
- Patients who performed MRI with DTI-FT study within 7 days before surgery;
- All the patients included in the study were newly diagnosed with glioma at their first surgery.

We excluded patients who did not agree to or could not undergo the functional MRI examination with DTI-FT or for whom the radiological examination could not be performed with the volumetric standards required for the analysis.

### Patient selection

We recorded clinical data such as age, gender, and clinical onset. Radiological information such as tumor site (identifying major lobe involvement, deep-seated or superficial location), tumor and peritumoral brain edema (PBE) volume, edema-tumor ratio, radiological and surgical morphology (distinguished between solid, cystic, or necrotic lesion) are reported.

Tumor grading are recorded distinguishing between high-grade (grade 4, isocitrate dehydrogenase (IDH) wild-type, HGG) and low-grade (grade less than 4 and IDH-mutated, LGG) tumors. Immunohistochemistry was performed, reporting ki-67%, EGFR expression status, MGMT, p53, ATRX and IDH. Surgical methods and the use of 5-ALA for eloquently and non-eloquently located tumors were previously and extensively reported elsewhere [3]. The extent of resection (EOR) was assessed by an experienced neuroradiologist in postoperative MRI within 48 h of surgery. Progression-free survival (PFS) and Overall survival (OS) were recorded in months.

Performance status was expressed using the KPS scale: such parameter was considered, as previously observed [3], as associated with OS. Specifically, it was recorded in four different moments: (1) Before surgery, (2) At 30 days after surgery (3) At the end of the adjuvant treatment, (4) At the last follow-up evaluation.

### Image acquisition

All the patients underwent a brain MRI scan, including a high field 3 Tesla volumetric study within 7 days before surgery with the following volumetric sequences: T2w, FLAIR, isotropic volumetric T1-weighted magnetization-prepared rapid acquisition gradient echo (MPRAGE) before and after intravenous administration of paramagnetic contrast agent and DTI with 3D tractography fiber tracking.

Volume of the contrast-enhancing lesion was calculated by drawing a region of interest (ROI) in a Volumetric enhancing post-contrast study weighted in T1 (a multi-voxel study), conforming to the margins of the contrast-enhancing lesion, using the free-hand assisted tool with software Horos (*LGPL license at*https://horosproject.org v3.3.6, *Annapolis*,* MD USA*) [17, 18]. PBE volume was calculated by drawing a ROI conforming to the hyperintense signal borders on the T2-weighted and Fluid Attenuated Inversion Recovery (FLAIR) 3D sequences and subtracting the previously calculated tumor volume. All volumes were measured in cm^3^ before anti-edematous therapy.

The relationship between tumor and brain edema was reported as the numerical ratio between the two values according to the formula:

$$\begin{aligned} &\frac{Tumor\;Volume\,(cm^{3}) + Edema\;Volume\,(cm^{3}))}{Tumor\;Volume (cm^{3}}\\&\;=Edema/tumor\;ratio\end{aligned}$$

DTI was acquired using a single-shot echo-planar imaging diffusion tensor sequences with equal settings (TR/TE = 7010/102 ms; FOV = 222 × 222 mm2; matrix 112 × 112; 50 slices without gap; slice thickness 2.7 mm; 32 non-collinear directions, b- value = 1000 s/mm^2^) using a dedicated head coil. Reconstruction with FT required for each image set at least one acquisition with 9 scalar volumes.

### Tractography

For DTI-FT the open-source validated [19] software DSI studio (https://dsi-studio.labsolver.org/) and BrainLab iPlan software (BrainLAB Inc., Feldkirchen, Germany) have been used. For the definition and evaluation of ROI feasibility metric analysis, we used these two tractography applications of different complexity to ensure cross-software validity.

MRI objects consisted of three volumes, manually contoured (slice by slice) with a ROI positioned manually. Two authors (D.A. and A.B.) who were blinded to any clinical or demographic patient information except the images measured the ROI volumes.

The seed ROI was placed outlining with the tool “free-hand drawing” region to be drawn freehand” the edges of the contrast-enhancing signal around the tumor. We defined the ROIs margin based upon the tracts’ obligatory pathways, derived from literature [20], own experiences in peritumoral tractography and following the limits of the BAT area.

Following the current clinical practice [11], the BAT was defined as the region adjacent to the gross tumor volume, which contains signal abnormalities on T2-weighted and FLAIR sequences. The minimum streamline length was set to 30 mm, and the maximum was set to 250 mm. The FT was filtered by the ROI at the evaluation. Unharmed and dislocated tracts were categorized as “unaffected”. Then, the FA mean, FA max, FA min, MD, and TI values within the volumes were extracted. The cutoff value for the FA to avoids false-positive and false-negative was placed specifically for the BAT at 0.05. The deviation for each value was less than 0.006, drawn by two authors on the two different software to ensure cross-validity.

### Statistical analysis

Statistics were performed using SPSS Statistics 25 (IBM, Armonk, NY, USA). Normality distribution was tested after D’Agostino-Pearson. Comparisons between nominal variables were made with the Chi-squared test. Comparisons between nominal and quantitative variables were made with t-students. The EOR means were compared with One-way and Multivariate ANOVA analysis, Contrast analysis, and Post-Hoc Tests. Continuous variable correlations have been investigated with Pearson’s Bivariate correlation. The threshold of statistical significance was considered *p* < 0.05.

## Results

### Population study

**Fig. 2:**
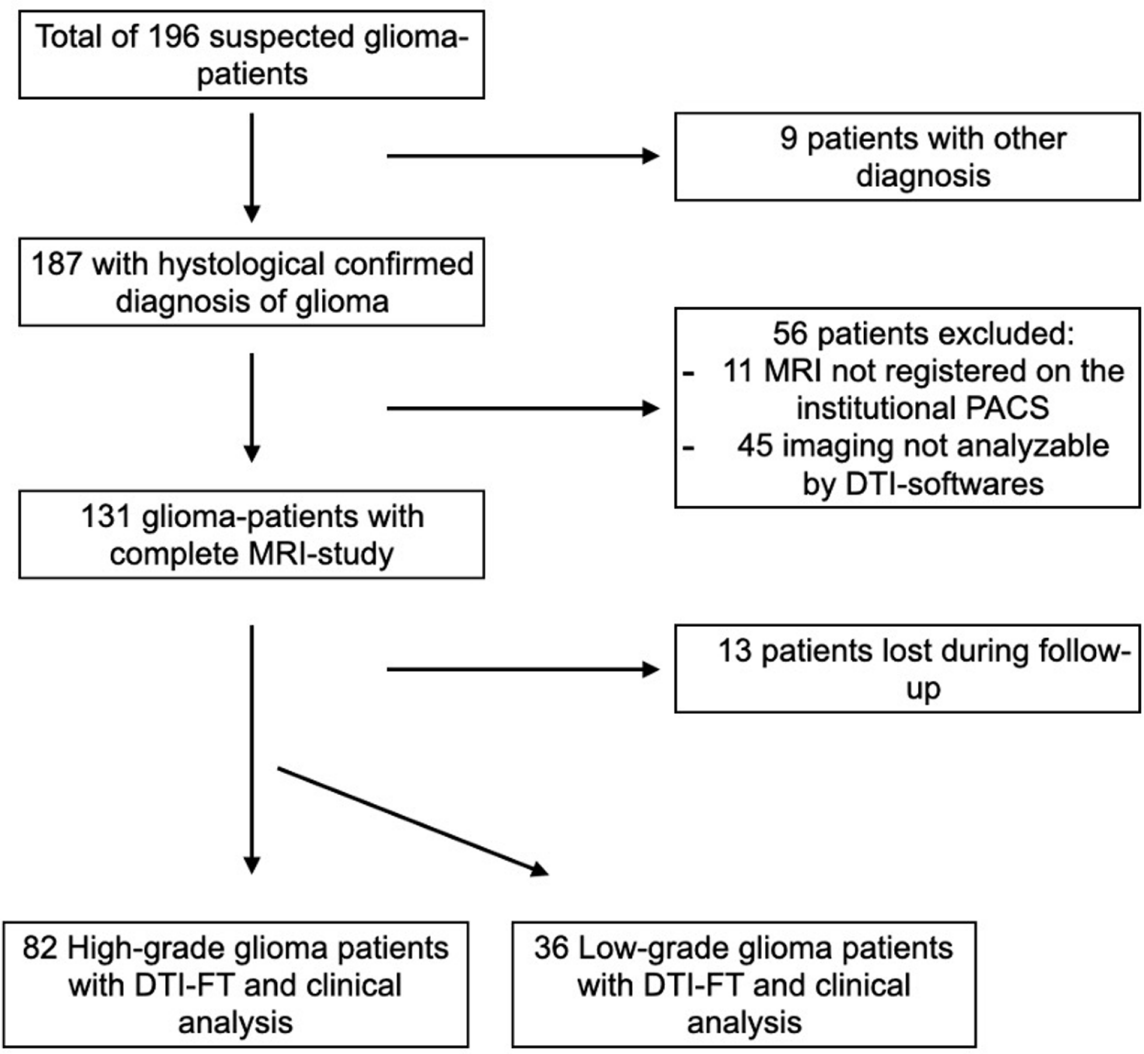
Flow-chart of the application of inclusion and exclusion criteria

196 patients underwent surgery for radiologically suspected intracranial gliomas. Applying inclusion and exclusion criteria, the final collection includes 118 patients (Fig. 2). The population consisted of 78 males (66.1%) and 40 females (33.9%), with a mean age of 60.6 years (min = 18, max = 80). 82 patients were found to have HGGs (WHO 4, 69.5%), and 36 patients had LGGs (WHO 1,2,3, 30.5%). All details on patient demographics, clinics and group analysis are summarized in Table 1.

### Radiological and clinical outcome

The mean tumor volume was 29.1 cm^3^ with no significant differences regarding grading (26.9 cm^3^ in HGGs, SD = 11.45 and 34.1 cm^3^ in LGGs, SD = 21.61, respectively, *p* = 0.22). The mean volume of PBE was 26.05 cm^3^, with a significant difference between HGG and LGG groups (28.8 cm^3^ SD = 2.42 versus 14.9 cm^3^ SD = 14.39, respectively, *p* = 0.05). There were no significant differences in tumor-edema ratio (310 versus 180, *p* = 0.25). The mean percentage of ki67 is 27%, with a significant difference between grading (35% in HGGs and 7.5% in LGGs, *p* < 0.001, respectively). EGFR is expressed in 18% of the population, and p53 was over-expressed in 25 patients (21.2%), both in the HGGs group. From the clinical outcome point of view, patients had a mean preoperative KPS of 85 with no significant difference in grading (85 for HGGs versus 90 for LGGs, *p* = 0.12). The mean KPS had significant differences between the two groups at postoperative, post-adjuvant therapy and at the last follow-up (65 versus 90), with HGGs presenting consistently lower values.

### Analysis of DTI metrics

We examined the values of FAmean, FA max, FA min, MD, and TI grade in the BAT, comparing it with the surgical series’ clinical, radiological, and outcome parameters.

**Fig. 3:**
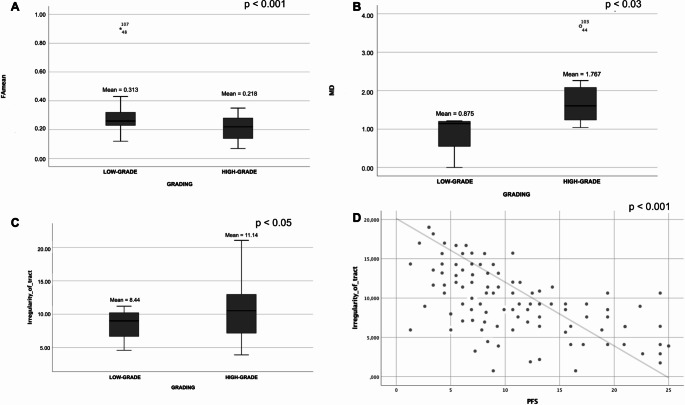
Q3

We identified that there is a significant inverse relationship between the FAmean value and grading (FA mean 0.313, SD = 0.11 for LGGs versus FA mean 0.218, SD = 0.007 for HGGs, *p* = 0.001), showing that a low-grade lesion is likely to result in more significant distortion/anisotropy of WM fibers than the aggressive HGGs (Fig. 3A).

In contrast, the relationship appears to be directly proportional regarding MD values (0.875 in LGGs, SD = 0.14 versus 1.767 in HGGs, SD = 0.71, *p* = 0.003, Fig. 3B) and TI values (8.44 in LGGs SD = 2.5 versus 11.14 in HGGs, SD = 2.2, *p* = 0.005, Fig. 3C).

In a multivariate analysis, FA mean, MD, and TI values are not influenced by the surgical site and tumor characteristics such as hemorrhage, necrosis, and cystic aspect (*p* = 1).

FA mean and MD values are susceptible to significant variations concerning tumor size and volume. Tumor volume correlate linearly with the value of FA (Pearson correlation= -0.360, *p* = 0.01). Edema volume correlate with the FAmean value (Pearson correlation = -0.351, *p* = 0.05) and MD value (Pearson correlation = 0.063, *p* = 0.05). In this evaluation, FA mean and MD values correlate with each other in a significant proportional manner (Pearson correlation=-0.516, *p* = 0.02).

### Specific analysis for TI parameter

TI showed an independent relationship with the degree of aggressiveness of the tumor regardless of tumor radiological features and dimensions, with a direct relationship with grading, ki67% (*p* = 0,05), and PFS (*p* < 0.001, Fig. 3D).

In LGGs, there is a significant relationship between TI value and EOR, with a higher percentage of GTR for higher TI values (Mean 11.2 SD = 1.93 for GTR versus Mean 8.9 SD = 2.13 for NTR, *p* < 0.01). This is probably because high TI values correspond to areas of altered WM texture and density capable of guiding the surgeon into marginal resection. WM enveloping the tumor and distorting surrounding bundles in terms of evident high TI values also has a significant clinical impact on tumor onset with seizures (patients with clinical onset of seizure have TI mean 14.46 compared to focal onset or incidental diagnosis with TI mean = 7.98).

We identified an optimal cutoff value for TI of 10, suggesting a higher risk of reduced PFS and KPS in patients with a score values > 9.

To evaluate whether the TI value ≥10 is an independent prognosis-related factor, we conducted an independent prognostic analysis for the performance status and PFS for the two groups (group TI < 10 and group TI≥10). The prognostic analysis used univariate and multivariate Cox regressions.

**Fig. 4:**
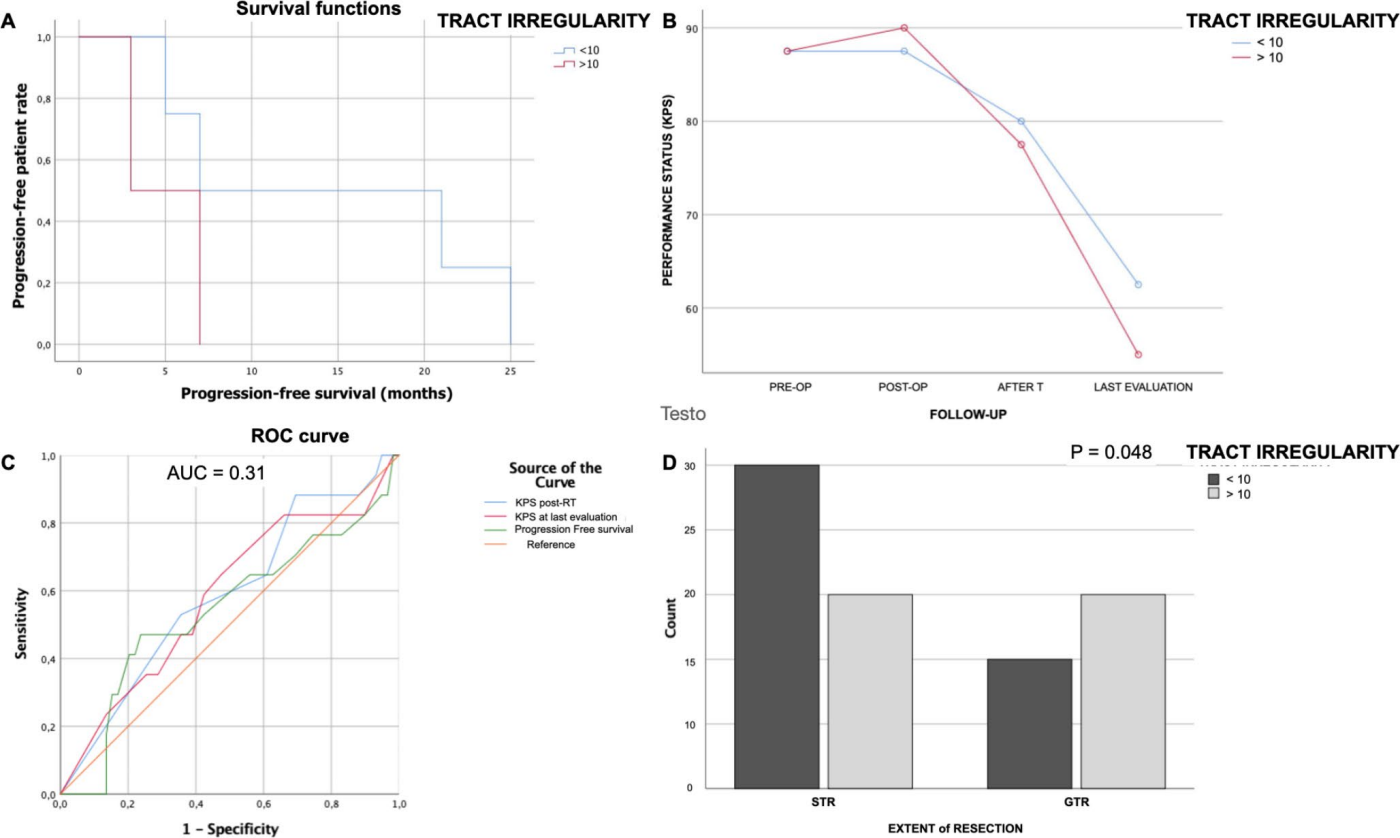
Q4

The univariate prognostic analysis showed that the value of TI with threshold 10 was an independent factor affecting the prognosis (*p* < 0.001). The multivariate prognostic analysis showed that the TI≥10 (*p* = 0.02), EOR (*p* < 0.001) and tumor grading (*p* = 0.04) were independent factors affecting the prognosis. In the TI≥10 group of patients, both univariate and multivariate prognostic analysis showed that the risk score was an independent factor affecting the PFS and KPS after surgery (Fig. 4A and B, *p* < 0.05). In the model values we obtained a cross-validated area under curve (AUC) of 0.31, CI 95% (*p* = 0.03, DeLong’s test) and single-validation AUC for KPS after RT (or at 6 months clinical evaluation for LGG without post-operative RT (95% CI, 0.4– 0.78), 0.583 (*p* = 0.03, DeLong’s test) for KPS at the last evaluation (95% CI, 0.44– 0.76), 0.541 (*p* < 0.001, DeLong’s test) and PFS (95% CI, 0.37– 0.709 *p* < 0.01, Fig. 4C).

Furthermore, performing a binomial analysis for the achievement of GTR between the two groups, we observed that in cases where total tumor resection was achieved, the TI value measured in the BAT more frequently showed a TI value ≥10. This difference was significant (Pearson chi-square *p* = 0.048) and could be dependent on greater tumor aggressiveness (with a high correlation with the presence of intratumoral necrosis, (Fig. 4D).

## Discussion

This study represents the first prospective clinical trial to track the treatment progress of glioma patients and assess the predictive significance of DTI measurements taken around the tumor. Our findings indicate that the mean FA value in the BAT of HGGs tends to decrease, while the MD value tends to rise. Additionally, we observed an inverse association between the mean FA value and grading. Further the new metric TI demonstrated an indipendent relationship with grading, ki67%, EOR, and PFS.

Histologically, it is known that tumor cell density decreases up to several centimeters from the macroscopic tumor volume [20, 21], and previous analyses [22] have shown that high fiber density values are inversely correlated with tumor cell number and tumor infiltration [23].

BAT stands for the area within the brain that is occupied by WM structures, and it is near the location of brain tumors. Typically, the fibers that cross this region are not part of the well-known and eloquent bundles that are frequently displaced or damaged by tumors. As a result, the values of anisotropy and diffusivity in BAT can have high variability, and it may be challenging to interpret its graphical representation [23, 24].

**Fig. 5:**
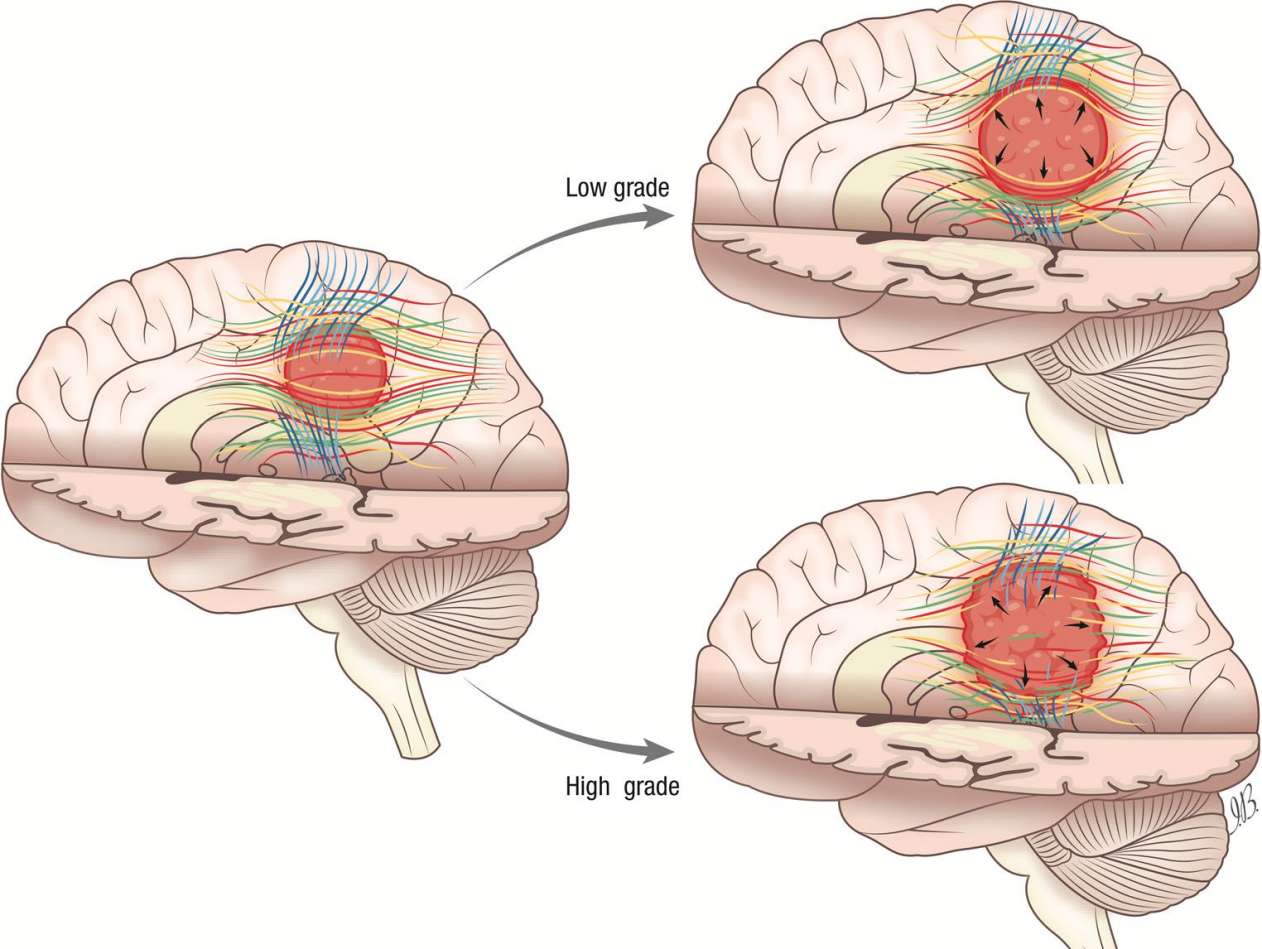
The illustration presents a schematic representation of the various ways in which glioma grading can distort the white matter (WM) of the brain adjacent tumor area (BAT), and how fractional anisotropy (FA), mean diffusivity (MD), and tumor intensity (TI) values can provide a quantitative definition. Our hypothesis suggests that the growth of a low-grade glioma (LGG) causes more significant distortion of WM fibers than the growth of a high-grade glioma (HGG). This can be demonstrated by an inverse relationship between the mean FA value and the grading. Additionally, an increase in MD can be attributed to an increase in extracellular spaces and the release of cellularity

We identified that the FA mean value tends to decrease in the BAT of HGGs (without ever reaching value = 0) while the value of MD tends to increase. These results confirm hypotheses [11, 24] that the FA mean tends to be significantly higher in the LGG than in the HGG, supposing this is linked to a greater heterogeneity of solid parts within the former (Fig. 5). Considering that a FA value of 0 represents completely unrestricted fiber diffusion, and a value of 1 signifies entirely directed diffusion in a single direction, the inverse relationship between the FA mean value and grading indicates that LGGs will likely lead to higher WM fiber anisotropy than HGGs [25] also in the BAT. In HGG, though there was the destruction of WM fibers causing the decrease of FA compared with normal-appearing WM, its value did not decrease to extremely low because the increase in cell density and vascularity gave directionality to the water diffusion in extracellular space, resulting in compensation of decreased FA (thus eliminating the possibility of obtaining a false negative response); on the contrary in LGGs, cells were loosely and randomly arranged in a fibrillary matrix, where water diffused almost freely in all direction thus leading to the significant increase of FA. Besides, increased MD would be correspondingly observed due to the increased extracellular spaces and decreased cellularity [26].

The main reliability problem of the DTI metric statistical descriptors [26] is that the extent of fiber density, directionality, and anisotropy is greatly affected by several variables, including tumor volume, PBE, and the tumor site location [27]. We show that glioma size and PBE could influence FA and MD values, and we confirm, in part, the results of the study by Kinoshita et al. [28] that reported an apparent diffusion coefficient in regions of tumor infiltration primarily affected these variables. Research on the combination of DTI and MR spectroscopy suggested that FA was uncorrelated and even contradictorily higher in LGGs [8]. At the same time, ADC value correlated significantly with histologic grading [28], and lower ADC indicated HGGs, concluding that DTI may not be helpful for preoperative differentiation precisely because of the presence of variable PBE volume [24, 29, 30].

This has led some researchers to detect other metrics that improve the study of tumor boundaries and infiltration [31]. With the shape metrics, Yeh FC [15] introduced an interesting quantification of WM tracts around a ROI to better investigate the shape characteristics of the human association pathways, allowing a deeper understanding of the fibers distortion in relation to tumor and edema volumes [7].

**Fig. 6:**
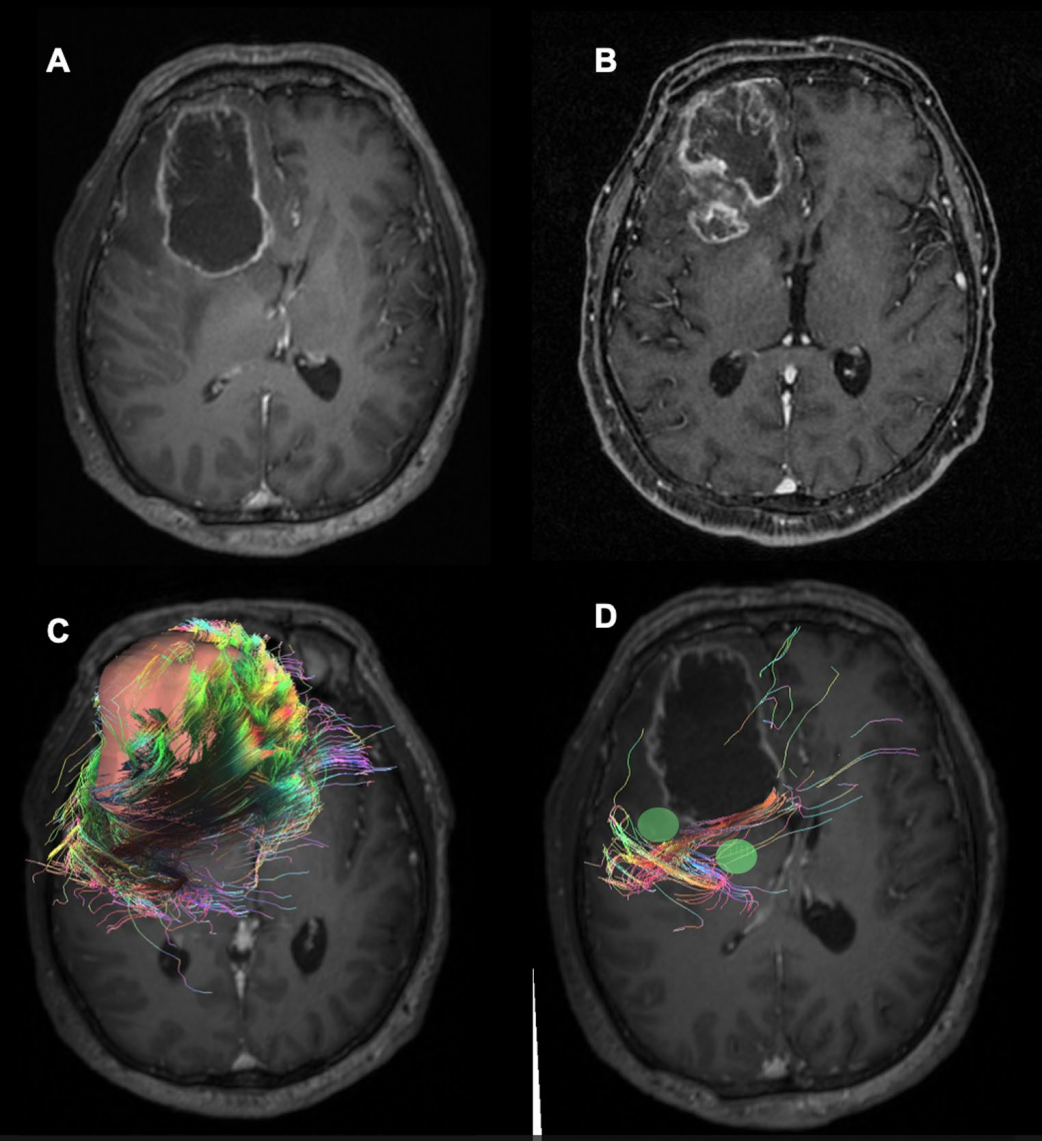
We present an illustrative case of a 57-year-old patient treated surgically with total excision of a right frontal glioblastoma (**A**) who experienced disease recurrence seven months after diagnosis (**B**). In a retrospective analysis of pre-operative DTI, we note low mean values of FA (mean 0.04), high mean values of MD (mean 0.45), and TI (mean 7.022) around the tumor (**C**). If we mark the areas of highest expression of TI (green areas), we observe that they are located right at the highest growth of recurrence and where the WM-fibers are most irregular (**D**)

Specifically, the TI value could be helpful for tumor grading and surgical planning [15]. TI demonstrated an independent relationship with the degree of aggressiveness of the tumor regardless of radiological features and dimensions, also showing a relationship with grading, ki67%, EOR, and PFS. It is remarkable that at higher TI values (with a threshold of 10), more patients in our series achieved a GTR. This suggests that areas around the lesion with high WM irregularity are more likely to be appreciated as macroscopically altered by the surgeon, who is more likely to proceed with resection. A high TI value corresponds to areas of altered WM consistency that favor the surgeon to provide a more extensive excision, thus obtaining GTR results more frequently. Furthermore, TI values correlate inversely with PFS, suggesting that it could be an indirect measure of microscopic tumor infiltration [31–33] (Fig. 6). In addition, the TI values exhibit an inverse relationship with PFS, which indicates that they may serve as an indirect measure of microscopic tumor infiltration. Although this is currently a preliminary assessment, the TI parameter may potentially be utilized as an intra-operative adjuvant marker in the future.

DTI-based functional neuronavigation could help planning aggressive resections of DTI-FT-defined abnormalities [34] and offering precise intraoperative imaging guidance to achieve a greater chance of improving progression-free and overall survival [35, 36].

### Further studies and limitations

The main limitation of this study is that it is a nonrandomized observational study with a limited number of patients. Although highly valued for its clinical applications, the validity of quantitative measurement of various DTI metrics is impaired by several factors: the different acquisition times performed during the MRI, the use of non-validated software, and the echo-planar imaging sequence used for DWI acquisition subjected to many artifacts. The best approach to determine the optimal cutoff value for FA in the context of BAT remains unknown. If an FA cutoff of 0.15 appears to be a reasonable choice for neurosurgical patients, potentially streamlining the process of tractography, it is essential to maintain control over infiltrating fiber bundles, which may necessitate lower cutoff settings [37].

Further, the tracking of an ROI in all imaging analysis software is done with a semi-automatic system of the free-hand drawing tool, which although it has good sensitivity in identifying the edge of a signal hyperintensity from contrast uptake remains a non-standardized method subject to a risk of bias.

## Conclusion

DTI-FT quantitative parameters measured in the BAT promise a quantitative value in preoperatively predicting patient outcomes and could be a valuable tool in surgical planning. In particular, TI, as a new parameter, appears to be an independent marker of tumor aggressiveness and expression of strong alteration of white matter bundles in the brain adjacent tumor area potentially allowing, once studied, to guide supramarginal surgical resection of glioma.

**Table 1:** Population study of the main clinical, surgical and radiological parameters evaluated for the study and analysis between patients diagnosed with high-grade glioma (HGG) and low-grade gliomas (LGG)

		Total patients (118)		High-grade (82)	Low-grade (36)	P-value
Gender	M	78	66.1%	58	18	
	F	40	33.9%	24	16	
Age	Mean	60.6		63.5	54	1
	Min-Max	18–80				
Lobe involvement	Frontal	68	57.6%	46	20	1
	Temporal	38	32.2%	30	8	
	Parietal	30	25.4%	20	8	
	Occipital	0	0%	0	0	
Location	Deep/periventricular	50	42.4%	38	12	0.9
	Superficial/convexity	68	57.6%	44	24	0.77
Morphology	Solid	67	56.7%	40	30	0.06
	Cystic	35	29.6%	28	6	1
	Necrotic	16	13.6%	14	0	
Hemorragic		8	6.8%			
Clinical debut	Focal deficit	37	31.4%	26	8	
	Seizure	37	31.4%	28	17	
	Cognitive deficit	35	29.7%	8	28	
	Incidental	9	7.6%	28	8	
Tumor volume	Mean (mm3)	29.1		26.9	34.1	0.22
Edema volume	Mean (mm3)	26.05		28.8	14.9	0.05
Tumor-edema ratio	Mean	1459		310	180	0.25
Extent of resection	GTR	53	44.9%	42	16	0.02
IDH-mutated		37	31.4%			
ki67%	Mean	27		35	7.5	< 0.01
EGFR expression		18	15.3%			
P53 expression		25	21.2%			
KPS (Mean)	Pre-operative	85		85	90	0.12
	Post-operative	80		80	90	0.03
	Post-RT	80		80	90	0.02
	Follow-up	65		65	90	< 0.01
PFS (Mean)		20		8	26	< 0.01
OS (Mean)		27		15	48	< 0.01
FA	Mean	0.248		0.218	0.313	0.01
	Min	0.1		0.1	0.12	0.6
	Max	0.53		0.51	0.53	0.6
MD		1.52		1.12	0.87	0.05
TI		10.41		11.14	8.44	0.05

## Data Availability

No datasets were generated or analysed during the current study.

## Abbreviations

CNS: Central nervous system
BAT: Brain adjacent Tumor area
WM: White matter
WHO: World Health Organization
GB: Glioblastoma
IDH: Isocitrate dehydrogenase
LGG: Low grade glioma
HGG: High grade gliomas
ATRX: Histone chaperoneαthalassemia mental retardation X-linked
OS: Overall survival
EOR: Extent of resection
5-ALA: 5-aminolaevulinic acid
WM: White matter
MRI: Magnetic resonance imaging
FLAIR: Fluid attenuated inversion recovery
DWI: Diffusion weighted imaging
DTI: Diffusion tensor imaging
DTI-FT: DTI Fiber tracking
CST: Cortico spinal tract
GQI: Generalized q-sampling imaging
ROI: Region of interest
KPS: Karnofsky performance scale
GTR: Gross total resection
fMRI: Functional MRI
FA: Fractional anisotropy
MD: Mean diffusivity
TI: Tract irregularity
PFS: Progression free survival
MPRAGE: Magnetization prepared rapid acquisition gradient echo
